# Prophylactic administration of ondansetron in prevention of intrathecal morphine-induced pruritus and post-operative nausea and vomiting in patients undergoing caesarean section

**DOI:** 10.1186/1471-2253-15-18

**Published:** 2015-02-17

**Authors:** Ram Bhakta Koju, Bandana Sharma Gurung, Yashad Dongol

**Affiliations:** Department of Anaesthesia and Critical Care, KIST Medical College, Imadol, Lalitpur, Nepal; Previously, Department of Anaesthesia and Critical Care, Patan Hospital, Patan, Lalitpur, Nepal; Department of Obstetrics and Gynaecology, KIST Medical College, Imadol, Lalitpur, Nepal; Department of Biochemistry, KIST Medical College, Imadol, Lalitpur, Nepal

**Keywords:** Caesarean section, Intrathecal morphine, Nausea and vomiting, Ondansetron, Opioids, Pruritus

## Abstract

**Background:**

Intrathecal morphine is commonly used for post caesarean analgesia. However, their use is frequently associated with the incidence of troublesome side effects such as nausea, vomiting and pruritus. Various mechanisms have been postulated for the opioid-induced pruritus, with a variety of medications with different mechanisms of actions formulated for the prevention and treatment. But, the results are inconsistent and hence the prevention and treatment of opioid-induced pruritus still remains a challenge. Ondansetron which is antiemetic, non-sedative and has no antianalgesic effect is an antagonist to 5-HT3 receptor, the receptor with which opioids interacts and imparts its effects. Ondansetron, thus, would be an attractive treatment strategy for both opioid-induced pruritus and post-operative nausea and vomiting.

**Methods:**

After the approval from institutional review committee and written consent received from the patient, 50 healthy parturients of ASA I and II physical status undergoing caesarean section under spinal anaesthesia were enrolled for the study. They were randomly categorized into placebo group (2 ml normal saline) and treatment group (2 ml of 4 mg ondansetron), each group containing 25 patients. Pruritus and post-operative nausea and vomiting scores were recorded up to 24 hours after the administration of intrathecal morphine. Statistical analysis was performed using chi-square test.

**Results:**

The incidence, severity and necessity of treatment for pruritus in the treatment group was significantly reduced compared to the placebo group (16% vs 88%). Similarly, the risk of post-operative nausea and vomiting in the treatment group was less compared to the placebo group (8% vs 56%).

**Conclusion:**

Prophylactic administration of ondansetron to parturients receiving intrathecal morphine for post-operative analgesia provides a significant reduction of intrathecal morphine-induced pruritus and nausea and vomiting.

**Trial registration:**

CTRI/2015/01/005362 registered on 07/01/2015 in Clinical Trials Registry – India (ctri.nic.in).

## Background

Neuraxial anaesthesia, which includes epidural anaesthesia and intrathecal anaesthesia, is a frequent anaesthetic approach for caesarean delivery and other lower abdominal and lower limb anaesthetic procedures. The addition of neuraxial morphine to local anaesthetics provides an effective and prolonged post-operative analgesia. Neuraxial administration of morphine - an opioid, which is considered as a gold standard for analgesia, has been associated with a frequent incidence of pruritus and post-operative nausea and vomiting (PONV) [1–3]. The incidence of PONV in patients who received an intrathecal opiate is 60% - 80% [4]. The incidence of neuraxial opioid-induced pruritus varies widely from 30% - 60% after orthopedic surgery with intrathecal morphine injection [3, 5–7] and from 60% - 100% in pregnant women after neuraxial opioid administration [3, 8–10]. Parturients appear to be the most susceptible to neuraxial opioid-induced pruritus which probably might be due to the interaction of oestrogens with opioid receptors [8, 11]. Although the exact mechanism of neuraxial opioid-induced pruritus is unclear, the postulated mechanisms include the presence of an “itch centre” in the central nervous system (CNS), medullary dorsal horn activation, antagonism of inhibitory transmitters, modulation of 5-hydroxytryptamine subtype 3 (5-HT3) or serotonergic pathways and the involvement of prostaglandins [3, 8]. There is dense concentration of opioid receptors and 5-HT3 receptors in the dorsal part of the spinal cord and the nucleus of the spinal tract of the trigeminal nerve in the medulla [8]. Activation of these receptors by neuraxial opioid administration or by circulating oestrogen in the parturients results in neuraxial opioid-induced pruritus which is usually localized to the face, neck, or upper thorax [7]. Nalbuphine, propofol and ondansetron have been used effectively in the treatment of pruritus associated with neuraxial morphine in surgical patients [12]. In our clinical setting, we undertook a prospective, randomized, double-blinded and placebo controlled study to assess the effectiveness of prophylactic intravenous (IV) administration of ondansetron in the prevention of intrathecal morphine-induced pruritus and PONV.

## Methods

This prospective, randomized, double-blinded and placebo controlled study was conducted at Patan Hospital, Patan, between August 17, 2008 to January 14, 2009. The institutional review committee of Patan Academy of Health Sciences (IRC-PAHS) approved the study protocol and written, informed consent was obtained from each patient. Parturients of American Society of Anesthesiologists (ASA) class I or II physical status scheduled for caesarean delivery under spinal anaesthesia were recruited in the study. Patients with known allergy to ondansetron, morphine or bupivacaine and those with pruritogenic systemic disease, a coexisting skin disorder or preexisting pregnancy-induced pruritus were excluded from the study. Similarly, patients with any contraindication for spinal anaesthesia or those who refused to participate in the study were also excluded.

Fifty patients were randomly divided into two groups - P group (placebo group, n = 25) and O group (treatment group, n = 25). P group received 2 ml of normal saline whereas O group received 2 ml of a 4 mg ondansetron IV injection. The study drug (i.e., 2 ml of 4 mg ondansetron) and the placebo (i.e., 2 ml of normal saline) were prepared by a nurse anaesthetist. She was oriented of the study procedure but neither involved in the study nor in the patient care. We used the ondansetron and normal saline supplied by the pharmaceuticals that were available in the hospital pharmacy. It was ascertained that the same manufacturer was used for either drugs in the study population. The study drugs were given 30 minutes before administration of spinal anaesthesia. Both the patients and the anaesthesiologists performing the spinal anaesthesia and collecting the post-operative data were blinded as to the study drugs. A nurse anaesthetist not involved in the study assisted in maintaining the randomization of sample in a double-blinded fashion, using a simple lottery method.

IV cannulation was done with 18 gauge cannula and patients were prehydrated with crystalloid solution 5-10 ml/kg. Spinal anaesthesia was performed at the level of L3-4 or L4-5 interspace with a 25 gauge Quincke-type spinal needle using 2.3 ml of 0.5% (11.5 mg) hyperbaric bupivacaine and 0.2 ml (0.2 mg) preservative-free morphine mixed in the same syringe. With constant monitoring of the non-invasive blood pressure, heart rate and urine output, hydration is maintained with crystalloid solution, intra-operatively and post-operatively for at least 24 hours. Post-operative wound pain was assessed with a 10-point visual analogue scale (VAS). Pethedine was used if patient complained of pain.

Post-anaesthesia care was provided as per the institutional monitoring protocol. Resident doctors – who were blinded observers - were involved in the patient care and data collection in the format provided. The onset of pruritus was assessed and recorded every 15 minutes for 4 hours along with the complaint by the patient. Pruritus scores were then evaluated at 4, 8 and 24 hours post-surgery. The degree of pruritis was categorized as 0 = no pruritus; 1 = mild pruritus; 2 = moderate pruritus; and 3 = severe pruritus. At the same time, patients were also evaluated for nausea and vomiting and categorized as 0 = no nausea or vomiting; 1 = mild nausea; 2 = intense nausea; 3 = vomiting. 10 mg intravenous metoclopramide was the drug of choice to treat vomiting or intense nausea (score ≥ 2). For patients with pruritus who requested the treatment, antihistamines such as pheniramine maleate and μ-opioid receptor antagonists such as naloxone were used depending upon the severity assessed by the clinician, if required.

A power analysis showed that 21 patients in each group would be sufficient to detect a difference of 60% pruritus incidence between the treatment and placebo group with a power of 95% and at 1% significance level. The perceived pruritus incidence difference between treatment and placebo group was derived from the study of Yeh et al. [9] where pruritus incidence in treatment group (p1) = 0.25 and pruritus incidence in placebo group (p2) = 0.85. Statistical analysis of the results was performed using chi-square test to compare the categorical variables. Data are also presented in mean with standard deviation and percentage.

## Results

All 50 patients were enrolled in the study during the period of five months and all the patients enrolled participated until the completion of the study. The demographic data of the patients involved in the study were tabulated in Table 1.

**Table 1:** **Demographic characteristics of the patients** ***(Mean ± SD)***

Variables	Study group	
	Placebo (P)	Treatment (O)
	n = 25	n = 25
Age (yrs)	25.2 ± 3.27	24.2 ± 3.56
Height (cm)	157.4 ± 4.77	155 ± 4.12
Weight (kg)	69.2 ± 9.96	70.8 ± 8.56

**Figure 1:**
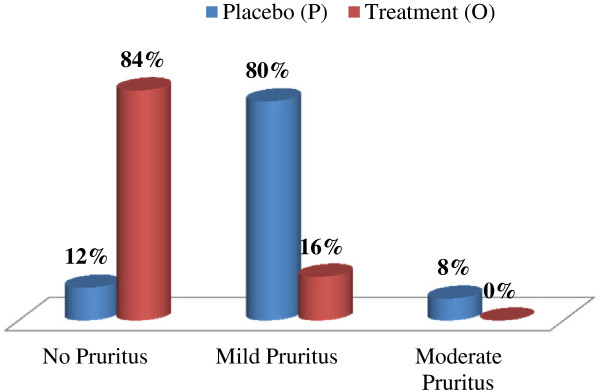
**Pruritus severity.**

**Figure 2:**
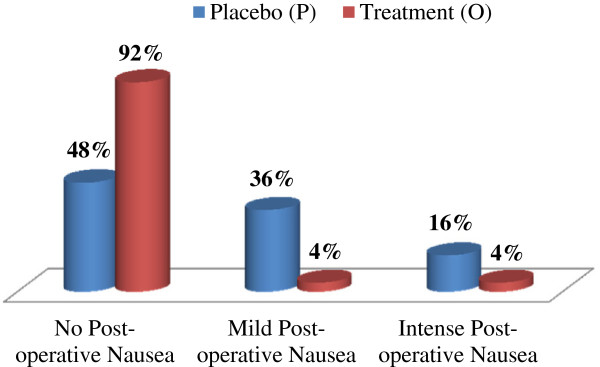
**Post-operative nausea severity.**

Post-surgery pruritus occurred in 88% of the patients who received the placebo injection whereas only 16% of the patients who received 4 mg of ondansetron prophylactically developed pruritus. Similarly, 56% of the patients in the placebo group complained of post-operative nausea whereas only 8% of the patients in the treatment group complained of post-operative nausea. The incidence of pruritus and post-operative nausea in the study groups are statistically significant (p < 0.001) and illustrated in Table 2. However, the onset and the duration of pruritus in both the groups, as shown in Table 3, were similar and statistically insignificant (P > 0.05). The difference in the severity of pruritus scores in both groups was statistically significant (P < 0.001) as shown in Figure 1. There were no patients with severe pruritus in either group. In the P group, 8% of cases (n = 2) had moderate pruritus, whereas no patient in the O group had moderate pruritus. However, in our study, none of the cases required any sort of medications to treat the pruritus.The incidence of post-operative nausea was also significantly different (P < 0.001) between the P group (56%) and O group (8%). The severity scores of post-operative nausea in both the groups were also statistically significant (P < 0.001) as shown in Figure 2. There were no cases of vomitting in either of the groups. O group had a smaller incidence of both mild and intense post-operative nausea (4% each) whereas in P group the incidence for mild post-operative nausea and intense post-operative nausea was higher at 36% and 16%, respectively. All patients with intense post-operative nausea were treated with 10 mg IV metoclopramide.

**Table 2:** **Incidence of pruritus and post-operative nausea after cesarean section in placebo and treatment group**

Variables	Study group		P-value
	Placebo (P)	Treatment (O)	
	n = 25 (%)	n = 25 (%)	
Post-operative nausea	14 (56%)	2 (8%)	< 0.001
Pruritus	22 (88%)	4 (16%)	< 0.001

**Table 3:** **Onset and duration of pruritus**

Variables	Study group		P-value
	Placebo (P)	Treatment (O)	
	Mean ± SD	Mean ± SD	
First occurrence of pruritus in hours (after admission of intrathecal morphine)	2.64 ± 0.456	2.96 ± 0.334	>0.05
Duration of pruritus (itching) in hours	12.82 ± 0.56	14.25 ± 0.50	>0.05

As per the institutional protocol for patient care, pethedine was our choice of treatment if the study population complained of unbearable pain. We did not have any case that needed pethedine to manage the pain complained by the patients during our study period, probably due to the analgesic property of intrathecal morphine. However, acetaminophen 500 mg per rectal suppository was used at times – 3 cases in O group and 5 cases in P group, during 24 hours of observation. The data on pain scores and pain management were not included in this study as the prime objective of the study was not pain management.

There were no patients who were hypotensive intra-operatively or post-operatively. It is because vitals are continuously monitored and managed immediately upon requirement. Similarly, there were no profuse bleeding cases that lead to hypotension. Besides, crystalloids were used, whenever required, with continuous monitoring of vitals. Therefore, data on vitals such as blood pressure and heart rate were not mentioned in the manuscript.

## Discussion

Morphine, an opioid, is an alkaloid constituent of opium. It is the dried latex obtained as natural product in opium poppy (Papaver somniferum). Morphine is an archetypal opioid which, in clinical medicine, is still considered as a mainstay of analgesic therapy used to relieve intense pain and suffering. It elicits analgesia by stimulating the opioid receptors, a G protein-coupled receptor (GPCR) highly expressed in the central nervous system [13].

Despite its beneficial use in the treatment of acute or chronic pain, morphine induces various side effects such as nausea, vomiting and more importantly pruritus upon neuraxial injection [11, 14]. Pruritus - an unpleasant and irritating sensation leading to scratching - is a common adverse effect of neuraxial morphine with the highest prevalence (up to 100%) associated with intrathecal morphine administration [11]. It is generally mild and localized to the face and trunk, but it can be severe and cause significant maternal discomfort [15]. Despite its frequent occurrence and practice of utilizing various pharmacological therapies including antihistamines, 5-HT3 receptor antagonists, opiate antagonists, propofol (hypnotic agent), non-steroidal anti-inflammatory drugs (NSAIDS) and anti-dopaminergic drugs, there are no consistently effective therapies established for opioid-induced pruritus [3, 11, 16].

Pruritus caused by opioids develops shortly after analgesia and the prevalence, onset time, duration and severity depends on the type, route and dosage of opioid used. Lipid soluble opioids, such as fentanyl and sufentanil invoke pruritus of shorter duration. The use of the minimum effective dose of such opioids and the addition of local anaesthetics seems to decrease the prevalence and the severity of pruritus. Pruritus induced by intrathecal administration of morphine is of longer duration and difficult to treat. Intrathecal administration of opioids reaches the peak concentrations in the cerebrospinal fluid (CSF) almost immediately compared to the epidural administration. In epidural administration of opioids, the rise to peak concentration in CSF is relatively delayed (10–20 minutes with fentanyl and 1–4 hours with morphine) [3]. Furthermore, the epidural space contains an excessive venous plexus which assists in extensive vascular reabsorption of opioids administered epidurally. Therefore, the side effect of opioids such as pruritus is more common and intense in intrathecal morphine administration than in epidural administration [17].

Though pruritus is considered as the most common side effect of neuraxial administration of opioids, with the reported incidence between 30% and 100%, the exact mechanism behind the neuraxial opioid induced pruritus is yet unclear [14]. It is probably not related to histamine release since antihistamines are ineffective in the therapy of pruritus caused by neuraxial morphine [18] and, as well, other opioids such as fentanyl and sufentanil that do not release histamine also cause pruritus when administered into the neuraxis [19]. Another theory proposes that opioid receptors that are located both supraspinally and at the spinal cord level are activated by morphine. The μ receptor is mainly responsible for pain modulation and some side effects, especially pruritus and nausea or vomiting which explains the antipruritic effects of μ antagonists such as nalbuphine and naloxone [12, 20]. Thirdly, pruritus from neuraxial opioids may also be related to the excitatory effects of opioids on the nocifensive and non-nocifensive neurons in the anterior and posterior spinal horns [21]. Propofol, which has an inhibitory effect on the dorsal horn of the spinal cord, may relieve such neuraxial opioid-induced pruritus [22]. Lastly, the evidences from various studies and clinical practice for the treatment of postoperative nausea, vomiting and pruritus have strongly proposed 5-HT3 receptor interaction by opioids as a probable mechanism [12, 14]. Fan has reported that morphine can activate 5-HT3 receptors by a mechanism independent of opioid receptors [23] which implies the direct stimulation of 5-HT3 receptors in the dorsal horn of the spinal cord and in the medulla by intrathecal morphine injection possibly leading to pruritus [12]. 5-HT3 receptors are abundant in the dorsal horn of the spinal cord and the spinal tract of the trigeminal nerve in the medulla [14]. 5-HT3 receptor antagonists, such as ondansetron, are effective in the prevention and treatment of PONV [24, 25]. Studies have also shown that 5-HT3 receptor antagonists significantly reduced the risk of pruritus compared to placebo whereas some studies showed no significant differences, thus creating the conflict regarding the efficacy of prophylactic 5-HT3 receptor antagonists in neuraxial opioid-induced pruritus prevention [4, 7, 9, 12, 14, 24, 26]. A metaanalysis by George et al. suggests that prophylactic 5-HT3 receptor antagonists were ineffective in reducing the incidence of pruritus but significantly effective in reducing the severity and the need for treatment of pruritus in parturients who received intrathecal morphine for cesarean delivery. They were also effective for the treatment of established pruritus. However, more studies are recommended to settle the conflict regarding the efficacy of prophylactic 5-HT3 receptor antagonists in neuraxial opioid-induced pruritus prevention [25]. We, thus, intended to assay in our study the efficacy of prophylactic administration of 5-HT3 receptor antagonist ondansetron in the prevention of intrathecal morphine induced pruritus and PONV. We chose the dose of 4 mg of ondansetron as it has been proven successful in the treatment of intrathecal morphine-induced pruritus [12, 27]. However, the dose of 4–8 mg or 0.1 mg kg^-1^ are also in practice [9, 14]. Additionally, other 5-HT3 antagonists, such as tropisetron, granisetron and dolasetron are also used [14].

This study showed that the incidence of pruritus after intrathecal morphine injection in patients undergoing cesarean delivery was frequent (88%) which can be prophylactically managed by IV administration of ondansetron, which was similar to the study done by Yeh et al. [9]. On the other hand, the incidence of PONV can, as well, be effectively managed prophylactically by injection of ondansetron. However, for those patients who developed pruritus, the onset and duration were similar in both groups. Although IV ondansetron significantly reduced the incidence of intrathecal morphine-induced pruritus, this complication still occurred in approximately 16% of the patients, which suggests that these patients might need other treatment regimens such as naloxones (opioid receptor antagonists) or propofol (antagonist to excitatory effect on the dorsal horn of the spinal cord). However, we limited our study to a single type of 5-HT3 antagonist i.e., ondansetron with a fixed dose of 4 mg. Hence, a study examining dose-dependent effects and with 5-HT3 antagonists of different potency would be worthwhile to conduct in the future in this population.

## Conclusion

Nausea, vomiting and pruritus are the common side effects of intrathecal morphine administration as a part of spinal anaesthesia for the patients undergoing caesarean delivery. However, it can be efficiently managed by IV administration of 4 mg ondansetron 30 minutes prior to intrathecal morphine injection. In our study, it significantly reduced the incidence, severity and the need for treatment of pruritus and post-operative nausea.

## Authors’ information

Dr. Ram Bhakta Koju is an Associate Professor and Head of the Department of Anesthesia and Critical Care at KIST Medical College. Dr. Bandana Sharma Gurung is an Associate Professor at the Department of Gynaecology and Obstetrics in KIST Medical College. Mr. Yashad Dongol is a Lecturer and Clinical Biochemist at the Department of Biochemistry in KIST Medical College.
